# Anti-inflammatory drugs as potential antimicrobial agents: a review

**DOI:** 10.3389/fphar.2025.1557333

**Published:** 2025-04-08

**Authors:** Onyedika Emmanuel Okpala, Johana Rondevaldova, Ladislav Kokoska

**Affiliations:** Department of Crop Sciences and Agroforestry, Faculty of Tropical AgriSciences, Czech University of Life Sciences Prague, Prague, Czechia

**Keywords:** bacterial infection, cellulitis, cystitis, dual-action drugs, inflammation, osteomyelitis, septic arthritis, single-drug therapy

## Abstract

The association and causal role of infectious agents in chronic inflammatory diseases have major implications for public health, treatment, and prevention. Pharmacological treatment of combined infectious and inflammatory diseases requires the administration of multiple drugs, including antibiotics and anti-inflammatory drugs. However, this can cause adverse effects, and therefore, dual-action drugs need to be developed. Anti-inflammatory drugs that have already shown antimicrobial properties appear to be promising candidates. NSAIDs, namely aceclofenac, diclofenac, and ibuprofen, were tested in clinical trials with patients diagnosed with uncomplicated urinary tract infections (UTIs) and cellulitis. The administration of ibuprofen, a drug tested in the highest number of studies, resulted in symptom resolution in patients with UTIs. Additionally, ibuprofen caused a high survival rate in mice infected with *Pseudomonas aeruginosa* and demonstrated potent *in vitro* antibacterial effects against *Bacillus cereus*, *Escherichia coli*, and *Staphylococcus aureus,* including methicillin-resistant *S. aureus* (MRSA) (MIC 0.625–2.5 mg/L). For most anti-inflammatory drugs, only data showing their *in vitro* and *in vivo* antimicrobial effects are available. Among these, auranofin caused a high survival rate in mice infected with *Enterococcus faecium, S. aureus,* and *Clostridioides difficile.* It also produced a strong *in vitro* growth-inhibitory effect against *Streptococcus agalactiae, S. pneumoniae, S. aureus, S. epidermidis, Bacillus subtilis, C. difficile, E. faecalis, E. faecium,* and *Mycobacterium tuberculosis* (MIC 0.0015–5 mg/L). Similarly, aspirin caused a high survival rate in *M. tuberculosis-*infected mice and strong to moderate *in vitro* activity against *E. coli*, *B. cereus*, *P*. *aeruginosa*, *Enterobacter aerogenes*, *Klebsiella pneumoniae* and *Salmonella choleraesuis* (MIC 1.2–5 mg/L). Moreover, topical application of celecoxib resulted in a high reduction in MRSA burden in mice. However, it only caused moderate *in vitro* effects against *S. epidermidis*, *S. aureus* and *Bacillus subitilis* (MIC 16–64 mg/L). These data suggest that certain non-steroidal anti-inflammatory drugs (NSAIDs) are promising drug candidates for the development of dual-action drugs for the potential treatment of combined infectious and inflammatory diseases such as tuberculosis, musculoskeletal infections and UTIs. Nevertheless, future clinical trials must be conducted to ascertain the antibacterial effect of these NSAIDs before their practical use.

## 1 Introduction

Clinical and epidemiological studies have suggested an association between infectious agents and chronic inflammatory disorder (Karin et al., 2006). For example, tuberculosis (TB), musculoskeletal infections (MSKIs) and urinary tract infections (UTIs) may trigger chronic inflammation, which may lead to severe tissue damage (Lew and Waldvogel, 2004; Zumla et al., 2015; Abraham et al., 2001). Combating these infections may require that patients be treated with a combination of antibiotics and anti-inflammatory drugs. However, multiple drug administration (known as polypharmacy) may lead to adverse health consequences due to drug-drug and drug-disease interactions (Patyar et al., 2011; Steinman et al., 2006; Field et al., 2004). For example, the administration of high doses of amoxicillin/clavulanate in combination with warfarin is associated with a higher risk of over-anticoagulation (Abdel-Aziz et al., 2016 **)**. There is also a concern that the interaction between non-steroidal anti-inflammatory drugs (NSAIDs) and antihypertensive drugs may increase the risk of acute kidney disease (Lapi et al., 2013). To minimise the severe side effects caused by polypharmacy, there is a prevalent need to develop dual-action drugs, which are compounds that combine two different desired pharmacological actions and possess dual mechanistic effects due to their targeting of different effector mechanisms (Patyar et al., 2011). Besides the decreased adverse effects, the use of such drugs can lead to improved medication efficacy (Patyar et al., 2011). For example, the administration of rivastigmine, a dual inhibitor of acetylcholinesterase and butyrylcholinesterase, demonstrated significantly greater responses of cognitive and behavioural functions in patients with dementia than the selective acetylcholinesterase inhibitors such as donepezil and galantamine (Ballard, 2002; Kandiah et al., 2017). Similarly, preclinical and clinical data demonstrated that bupropion, an antidepressant medication used to treat depression, acts through the dual inhibition of norepinephrine and dopamine reuptake. Besides its efficacy, which is comparable to other antidepressants, bupropion therapy is not associated with common antidepressant-associated side effects, such as sexual dysfunction, weight gain, and sedation (Stahl et al., 2004). Romosozumab is an osteoanabolic drug with dual action used for the treatment of osteoporosis. It binds to and inhibits sclerostin (a natural inhibitor of bone formation) and exhibits a dual effect by stimulating bone formation and reducing bone resorption. Thus, romosozumab can be best characterised as a dual agent, demonstrating osteoanabolic and antiresorptive functions (Tabacco and Bilezikian, 2019). The case of romosozumab can also clearly illustrate the future market potential of dual-action drugs. For example, the sales of romosozumab increased 36% year-over-year to $431 million in the fourth quarter of 2024 and 35% for the full year (Amgen, 2024). Moreover, clinical experts suggest that the market share of romosozumab will increase each year until 2026/2027 when it reaches 50% of the eligible population (National Institute for Health and Care Excellence, 2022). Additionally, 12 months of treatment of osteoporosis patients with romosozumab followed by 4 years of alendronate was a more cost-effective option with greater quality-adjusted life years compared to other alternatives (Tabacco and Bilezikian, 2019; Amgen Canada Inc, 2022). Latanoprostene bunod, used for the treatment of glaucoma, is another example of a dual-action drug with great market potential. This agent increases trabecular and uveoscleral outflow and lowers intraocular pressure much better than other prostaglandin analogues (Mendelsohn, 2022). The drug, which recorded 18% total prescription growth in the fourth quarter of 2022, was launched in 15 countries in 2022 and was set to expand to 10 more by 2023 (Bausch and Lomb, 2022).

Another promising alternative to polypharmacy is the use of multi-target drugs, which simultaneously act on multiple pathways. This approach offers safer and more effective treatment options for complex diseases such as cancer, inflammation, diabetes, and central nervous system disorders (Brown and Superti-Furga, 2003; Kamb et al., 2007; Cavalli et al., 2008). For example, imatinib is an effective multi-target drug used for the treatment of chronic myelogenous leukaemia and gastrointestinal stromal tumours. This drug specifically targets proto-oncogene c-Kit, tyrosine-protein kinase ABL1, and platelet-derived growth factor receptors involved in cancer signalling (Slomovitz et al., 2004). Other strategies include targeted drug delivery systems, drug repurposing and deprescribing. A targeted drug delivery system facilitates the delivery of drugs to their specific target site in the body, thereby enhancing therapeutic effects with reduced adverse effects (Rayaprolu et al., 2018; Vargason et al., 2021). Nanocarriers are promising targeted systems that selectively and effectively deliver drugs to targeted sites through enhanced permeability and retention (Dang and Guan, 2020). Additionally, implantable local delivery carriers used for the treatment of osteomyelitis deliver antibiotics in a controlled, slow and sustained manner from an implant to the infection site (Smith et al., 2022). On the other hand, drug repurposing involves finding new therapeutic uses for existing drugs outside the scope of their original medical indication (Pinzi et al., 2024). This approach reduces risks as the compounds have already passed toxicity and safety studies (March-Vila et al., 2017). It also creates the opportunity for repurposing the already-in-use drugs for a second indication, thereby providing treatments for unmet medical needs (Pinzi et al., 2024). For example, sildenafil developed as an anti-hypertensive and anti-anginal drug (Ghofrani et al., 2006), is repurposed for the treatment of erectile dysfunction (Ashburn and Thor, 2004). Deprescribing is the supervised discontinuation of a drug (Reeve et al., 2015) to reduce overtreatment and prevent adverse effects (Wu et al., 2021; Page et al., 2016). For example, lowering the doses of proton pump inhibitors was more beneficial because it reduces the risk of side effects and drug interactions and potentially reduces the cost of drugs (Farrell et al., 2017). Despite these alternative therapeutic approaches to polypharmacy, dual-action drugs have not been fully developed for the treatment of combined infection-inflammatory conditions such as MSKIs, TB, and UTIs.

MSKIs are inflammatory conditions that affect the bones and joints, causing serious morbidity and posing significant management challenges (Arkader et al., 2016). Osteomyelitis and septic arthritis are the most severe forms of MSKIs (Colston and Atkins, 2018), and *Staphylococcus aureus* together with *S. epidermidis* are their primary causative agents (Kavanagh et al., 2018; Goldenberg, 1998). Other bacteria such as *Escherichia coli, Haemophilus influenzae, Neisseria gonorrhoeae, N. meningitidis, Pseudomonas aeruginosa, Proteus mirabilis,* and *Streptococcus pneumoniae* can also be involved in MSKIs (Goldenberg, 1998; Tarkowski, 2006). Epidemiological studies showed that the total osteomyelitis prevalence increased to 10.44% in Germany from 2008 to 2018 (Walter et al., 2021), while 16, 382 emergency visits were recorded in the USA in 2012 for septic arthritis (Singh and Yu, 2018). Additionally, it is estimated that the cost of osteomyelitis treatment may rise to € 500,000,00 per case (Hogan et al., 2013). Although septic arthritis and osteomyelitis are primarily caused by microbial infections (Kalinka et al., 2014; Beck-Broichsitter et al., 2015; Goldenberg, 1998), pre-existing inflammatory conditions such as rheumatoid arthritis (RA) are one of the most common risk factors associated with these diseases (Krasselt et al., 2021; Dinescu et al., 2021). Certain cases of MSKIs are also characterised by early progressive inflammatory destruction of bone, contributing to the disease pathogenesis (Lew and Waldvogel, 2004). Therefore, the efficient treatment of MSKIs may require co-administration of antibiotics and NSAIDs. For example, antibiotics co-administered with anti-inflammatory drugs to patients with chronic osteomyelitis caused complete symptom resolution (Kudva et al., 2019). Antibiotic therapy (e.g. vancomycin) and surgical debridement are the most common treatment options for septic arthritis and osteomyelitis (Lew and Waldvogel, 2004; Copley, 2009; Kolinsky and Liang, 2018). However, the administration of antibiotics may cause adverse reactions such as phlebitis, hypotension, ototoxicity, nephrotoxicity, hypersensitivity, neutropenia, and interstitial nephritis (Bruniera et al., 2015). Surgical debridement involves the removal of the necrotic bone and tissue to improve the infected local environment and enhance antibiotic delivery (Urish and Cassat, 2020). Since traumatic surgical debridement may cause pain and inflammation (Lima et al., 2014), NSAIDs such as naproxen may be administered to patients with the aim of minimising inflammatory reactions (Chen et al., 2018).

TB is a severe communicable chronic disease caused by *M. tuberculosis* (Lawn and Zumla, 2011; Spitaleri et al., 2019), with a higher rate of infection found in children and the elderly (Esmail et al., 2018; Tahan et al., 2020). This disease remains a serious threat to global health, with an increase in morbidity and mortality worldwide (Mahmoud et al., 2016; Sood et al., 2016). Yearly, about 1.8 million people die from TB (Sasindran and Torrelles, 2011). During the primary stage of TB infection, the bacteria replicate in the lungs, causing inflammation by attracting monocytes and other inflammatory cells (Sasindran and Torrelles, 2011). Thus, inflammatory responses in patients are the most common pathological characteristic of *M. tuberculosis* (Liu et al., 2018). Moreover, the emergence of multidrug-resistant TB exacerbated the spread of infection (Richeldi et al., 2004). The recommended treatment for drug-susceptible TB may include a 4-month regimen of rifapentine (belonging to rifamycins), isoniazid, pyrazinamide, and moxifloxacin (Carr et al., 2022). However, the rise in multidrug-resistant TB (TB resistant to isoniazid and rifampicin) has led to the search for new anti-TB drugs (Svensson et al., 2015). Moreover, pathologic host immune reactions and excessive inflammation, which may result in tissue damage, are responsible for treatment failure in TB patients (Zumla et al., 2015). Thus, NSAIDs have been recommended as host-directed therapy (Ivanyi and Zumla, 2013), which can act on host immune effectors to decrease host-destructive pathology, including inflammation (Kroesen et al., 2017).

UTI is the most common bacterial infection in women, and half of the female population develops UTIs at least once in their lifetime (Colgan and Williams, 2011). Uncomplicated UTIs, such as cystitis, affect people with no structural urinary tract abnormalities (Hooton, 2012; Nielubowicz and Mobley, 2010; Hannan et al., 2012), while complicated UTIs affect people with conditions that may compromise their immune defence systems, such as renal transplantation, pregnancy, and indwelling catheters (Lichtenberger and Hooton, 2008; Levison and Kaye, 2013). Although microorganisms such as *Klebsiella pneumonia* (6%), *Staphylococcus saprophyticus* (6%), *Enterococcus* spp (5%), and *P. mirabilis* (2%) may also be involved, *E. coli* (75%) is the main cause of UTIs (Flores-Mireles et al., 2015). The expression of type 1 fimbriae virulent factor, which promotes strong adherence of *E. coli* to the uroepithelium, may cause inflammation of the urinary tract (Abraham et al., 2001). Moreover, a previous report showed that this factor modulates inflammatory responses of the host immune cells by binding to neutrophils, macrophages, and lymphocytes, thereby triggering the release of inflammatory mediators (Abraham et al., 1999). Therefore, the anti-inflammatory effect of NSAIDs on the urothelium may help alleviate the symptoms of uncomplicated UTIs (Sachdeva et al., 2021). For example, a previous study showed that administering NSAIDs to patients with recurrent cystitis resulted in positive clinical outcomes (Chung, 2016). Since evidence indicates that certain NSAIDs have produced antimicrobial action in various *in vitro*, *in vivo* studies and produce symptom resolution in clinical experiments (Thangamani et al., 2015; Dutta et al., 2007a; Cassetta et al., 2014; Chan et al., 2017; Abutaleb and Seleem, 2020a; Bleidorn et al., 2010), they are considered good candidates for developing dual-action drugs for the treatment of UTI and other inflammation-associated infections, including TB and MSKIs.

Anti-inflammatory drugs are among the most used therapeutic groups of agents worldwide (Domingos et al., 2019). They are used for a wide variety of indications, including pain treatment, traumatism, inflammatory and autoimmune diseases, and many of them can be obtained over the counter (Gomez-Acebo et al., 2018). Corticosteroids and NSAIDs are the two main groups of anti-inflammatory drugs. Corticosteroids are steroid hormones produced physiologically by vertebrates and their synthetic analogues (Ferrara et al., 2019), which inhibit the phospholipase A2 enzyme (Whittle, 2000). They exhibit potent anti-inflammatory and immunosuppressive effects (Pallio et al., 2016; Stanbury and Graham, 1998). Although certain corticosteroids (e.g., corticosterone) exhibit some antibacterial effects, only a few of them produce this action which is inferior to the current antibacterial drugs (Dogan et al., 2017). On the other hand, the antimicrobial activities of NSAIDs have been demonstrated in a number of *in vitro* and *in vivo* studies (Zhang et al., 2021; Thangamani et al., 2015). Their anti-inflammatory mechanism of action is through the inhibition of cyclooxygenases (COX-1 and COX-2), the key enzymes involved in prostaglandin synthesis (Bindu et al., 2020; Yao and Narumiya, 2019). According to the WHO List of Essential Medicines, NSAIDs are among the most frequently prescribed anti-inflammatory drugs (Bindu et al., 2020). In addition, they are also used as antipyretics to reduce fever and as analgesics in pain management (Bindu et al., 2020; Yao and Narumiya, 2019). Evidence has shown that apart from taking antibiotics, patients are usually administered anti-inflammatory drugs to manage inflammation associated with several diseases. Thus, NSAIDs have become first-choice drugs for this purpose (Nugrahani et al., 2023). Because of the rich evidence of their antimicrobial effects (Thangamani et al., 2015; Chan et al., 2017), they are promising candidates for developing drugs with dual anti-inflammatory and antimicrobial activities to potentially treat combined infectious and inflammatory diseases such as MSKIs, TB and UTIs. Most importantly, using NSAIDs as a single-drug therapy could reduce the risk of adverse drug reactions caused by multiple drug co-administration. However, the results of current research on the antimicrobial effects of these drugs have yet to be sufficiently reviewed. Thus, this review summarises and critically analyses the *in vitro*, *in vivo*, and clinical data on the antimicrobial efficacy of anti-inflammatory drugs.

## 2 Antimicrobial activities of anti-inflammatory drugs

### 2.1 *In vitro* studies

The literature analysis identified 17 anti-inflammatory drugs, namely aspirin, auranofin, bromfenac, carprofen, celecoxib, diacerein, diclofenac, flufenamic acid, flurbiprofen, ibuprofen, indomethacin, meclofenamic acid, naproxen, nimesulide, sodium salicylate, tolfenamic acid and vedaprofen producing *in vitro* antimicrobial activity against 60 bacterial and fungal species including Gram-positive bacteria (*Bacillus anthracis, Bacillus cereus, Bacillus subtilis, Clostridioides difficile, E*. *durans, E. faecalis, E. faecium, Gemella haemolysans, Listeria monocytogenes, Micrococcus luteus, Mycobacterium smegmatis, M. tuberculosis, S*. *aureus, S. capitis, S. epidermidis, S. haemolyticus, S. hominis, S. intermedius, S. xylosus, Streptococcus agalactiae, S. mitis, S. pneumoniae, S. salivarius,* and *S. sanguinis*) and Gram-negative bacteria (*Acinetobacter baumannii, A. baylyi, Enterobacter aerogenes*, *E. cloacae*, *E. coli*, *Francisella novicida, F. tularensis, Helicobacter pylori*, *K*. *pneumoniae, N. gonorrhoeae*, *Paracoccus yeei, P. aeruginosa, Salmonella choleraesuis, S. typhi,* and *S*. *typhimurium*) and fungi (*Aspergillus brasiliensis, A. fumigatus, Candida albicans, C. glabrata, C. guilliermondi*, C. *krusei, C. lusitaniae, C. neoformans, C. parapsilosis, C. tropicalis, Cryptococcus gattii, C. neoformans, Epidermophyton fluccosum, Microsporum canis, M. fulva*, *M. gypseum*, *Trichophyton interdigitale, T. mentagrophytes, T. rubrum*, *T. tonsurans* and *T. violaceum*). Detailed data on the *in vitro* antimicrobial activity of anti-inflammatory drugs, including their MIC and MBC values, are shown in Table 1. Aspirin, auranofin, celecoxib, diclofenac, diacerein, and ibuprofen belong to the most frequently tested and antimicrobially active anti-inflammatory drugs.

**TABLE 1:** *In vitro* antimicrobial activity of anti-inflammatory drugs.

Compound	Microorganism	MIC (mg/L)	MBC (mg/L)	References
Aspirin	*Bacillus cereus*	2.5	5	Chan et al. (2017) Al-Bakri et al. (2009)
	*Candida albicans*	2.65	5.28	
	*Enterobacter aerogenes*	5	5	Chan et al. (2017)
	*Escherichia coli*	≥1.2	≥4.9	Al-Bakri et al. (2009), Chan et al. (2017)
	*Klebsiella pneumoniae*	5	5	Chan et al. (2017)
	*Pseudomonas aeruginosa*	2.03–5	≥4.8	Al-Bakri et al. (2009), Chan et al. (2017)
	*Salmonella choleraesuis*	5	5	Chan et al. (2017)
	*Staphylococcus aureus* ^a^ (MRSA standard strains and clinical isolates)	2.5–780	2.5–5	Chan et al. (2017), Ozturk et al. (2021)
Auranofin	*Acinetobacter baumannii* ^a^ (MDR, standard strain, urine and CNS isolates)	≥16	—	Harbut et al. (2015), Quadros Barse et al. (2024)
	*Bacillus subtilis*	0.05–0.5	—	Harbut et al. (2015), Quadros Barse et al. (2024)
	*Candida albicans*	1–16	—	Thangamani et al. (2017)
	*Candida glabrata*	8	—	
	*Candida neoformans*	0.5–4	—	
	*Candida parapsilosis*	4	—	
	*Candida tropicalis*	4–16	—	
	*Cryptococcus gattii*	0.5–8	—	
	*Clostridioides difficile*	0.25–4	—	Abdelkhalek et al. (2019), Abutaleb and Seleem (2020b)
	*Escherichia coli*	8–64	—	Thangamani et al. (2016a)
	*Enterococcus faecalis* ^a^ (VRE standard strains and clinical isolates of VRE from urine samples)	0.125–1	—	Harbut et al. (2015), Thangamani et al. (2016a), Abdelkhalek et al. (2018), Abutaleb and Seleem (2020a)
	*Enterococcus faecium* ^a^ (VRE, clinical isolates of VRE, resistant to ERY, TET, AMP, GEN, STR, TEIC)			
	*Klebsiella pneumoniae*	256	—	Thangamani et al. (2016a)
	*Mycobacterium tuberculosis*	0.5	—	Harbut et al. (2015)
	*Pseudomonas aeruginosa*	≥256	—	Thangamani et al. (2016a) Quadros Barse et al. (2024)
	*Salmonella Typhimurium*	128	—	
	*Streptococcus agalactiae*	0.0015–0.0625	—	
	*Streptococcus pneumoniae*	0.25	—	
	*Staphylococcus aureus* ^a^ (MRSA, MDR, drug-resistant clinical isolates from pacemaker, osteomyelitis, CVC)	0.0625–1.357	—	Cassetta et al. (2014), Harbut et al. (2015), Thangamani et al. (2016a), Chiaverini et al. (2022), Quadros Barse et al. (2024), Ferretti et al. (2025)
	*Staphylococcus epidermidis* ^a^ (drug-resistant clinical isolates from pacemaker and CVC), standard strain (biofilm producer)	0.0625–0.25	—	Cassetta et al. (2014),Chiaverini et al. (2022), Thangamani et al. (2016b)
Bromfenac	*Acinetobacter baylyi*	1,670	—	Yin et al. (2014)
	*Bacillus subtilis*	418	—	
	*Escherichia coli*	835	—	
	*Staphylococcus aureus*	835	—	
Carprofen	*Acinetobacter baylyi*	340	—	Yin et al. (2014)
	*Bacillus subtilis*	85	—	
	*Escherichia coli*	680	—	
	*Staphylococcus aureus*	85	—	
Celecoxib	*Bacillus anthracis*	16	—	Thangamani et al. (2015)
	*Bacillus subtilis*	16	—	
	*Francisella tularensis*	16	—	Chiu et al. (2009)
	*Francisella novicida*	32	—	
	*Listeria monocytogenes*	32	—	Thangamani et al. (2015)
	*Mycobacterium smegmatis*	16	—	
	*Staphylococcus aureus* ^a^ (MRSA standard strains and clinical isolates, VRSA)	16–64	—	Chiu et al. (2012), Thangamani et al. (2015), Gajdacs and Spengler (2019) Okpala et al. (2024)
	*Staphylococcus epidermidis*	16	—	Chiu et al. (2012)
	*Streptococcus pneumoniae*	64	—	Thangamani et al. (2015)
Diacerein	*Enterococcus durans*	128	—	Zhang et al. (2019)
	*Enterococcus spp*	8–32	—	
	*Gemella haemolysans*	128	—	
	*Micrococcus luteus*	128	—	
	*Staphylococcus aureus* ^a^ (MRSA standard strains and clinical isolates)	4–64	—	Nguon et al. (2013), Zhang et al. (2019)
	*Staphylococcus capitis*	32	—	
	*Staphylococcus epidermidis*	1–16	—	
	*Staphylococcus haemolyticus*	4–16	—	
	*Staphylococcus hominis*	8	—	
	*Staphylococcus intermedius*	4–16	—	
	*Staphylococcus xylosus*	2–16	—	
	*Streptococcus sanguinis*	32–128	—	
	*Streptococcus salivarius*	128	—	
	*Streptococcus mitis*	128	—	
	*Streptococcus pneumoniae*	64–128	—	
Diclofenac	*Bacillus cereus*	1.25	2.5	Chan et al. (2017)
	*Mycobacterium tuberculosis*	10–25	40	Dutta et al. (2007a)
	*Staphylococcus aureus*	0.3125–400	≥2.5	Dastidar et al. (2000), Chan et al. (2017), Zhang et al. (2021), Ozturk et al. (2021) Alves de Lima e Silva et al. (2021)
	*Staphylococcus epidermidis*	125	—	Zhang et al. (2021)
Flufenamic acid	*Acinetobacter baylyi*	1,400	—	Yin et al. (2014)
	*Bacillus subtilis*	88	—	
	*Neisseria gonorrhoeae*	2–8	—	Seong et al. (2020)
	*Staphylococcus aureus*	175	—	Yin et al. (2014)
Flurbiprofen	*Candida albicans*	64	—	Chowdhury et al. (2003)
	*Epidermophyton fluccosum*	32	—	
	*Microsporum canis*	32	—	
	*Microsporum gypseum*	32	—	
	*Microsporum fulva*	64	—	
	*Trichophyton mentagrophytes*	16	—	
	*Trichophyton rubrum*	32	—	
	*Trichophyton tonsurans*	32	—	
	*Trichophyton interdigitale*	32	—	
	*Trichophyton violaceum*	32	—	
Ibuprofen	*Bacillus subtilis*	2.5–5	—	Al-Janabi (2010)
	*Bacillus cereus*	0.625	2.5	Chan et al. (2017)
	*Candida albicans*	2	—	Pina-Vaz et al. (2000)
	*Candida glabrata*	3	—	
	*Candida krusei*	1–3	—	
	*Candida tropicalis*	3	—	
	*Candida guilliermondi*	1–2	—	
	*Candida lusitaniae*	2–3	—	
	*Enterobacter aerogenes*	5	—	Al-Janabi (2010)
	*Escherichia coli*	2.5–5	—	
	*Enterobacter cloacae*	5	—	
	*Helicobacter pylori*	125	250–500	Shirin et al. (2006)
	*Paracoccus yeei*	1.25–5	—	Al-Janabi (2010)
	*Pseudomonas aeruginosa*	512	—	Chen et al. (2023)
	*Staphylococcus aureus* ^a^ (MRSA standard strains and clinical isolates)	1.25–2000	≥2.5	Chan et al. (2017), Oliveira et al. (2019), Al-Janabi (2010), Ozturk et al. (2021), Tabatabaeifar et al. (2022)
	*Salmonella typhi*	2.5–5	—	Al-Janabi (2010)
Indomethacin	*Helicobacter pylori*	100	62.5–125	Shirin et al. (2006)
Meclofenamic acid	*Neisseria gonorrhoeae*	4–32	—	Seong et al. (2020)
Naproxen	*Staphylococcus aureus*	780	—	Ozturk et al. (2021)
Nimesulide	*Aspergillus fumigatus*	770	—	de Matos et al. (2017)
	*Cryptococcus gattii*	62	—	
	*Cryptococcus neoformans*	62	—	
	*Epidermophyton floccosum*	112	—	
	*Microsporum canis*	112	—	
	*Trichophyton mentagrophytes*	≥2	—	
	*Trichophyton rubrum*	160	—	
Sodium salicylate	*Helicobacter pylori*	4,000	—	Shirin et al. (2006)
Tolfenamic acid	*Acinetobacter baylyi*	1,300	—	Yin et al. (2014)
	*Bacillus subtilis*	82	—	
	*Neisseria gonorrhoeae*	2–8	—	Seong et al. (2020)
	*Staphylococcus aureus*	163	—	Yin et al. (2014)
Vedaprofen	*Acinetobacter baylyi*	705	—	Yin et al. (2014)
	*Bacillus subtilis*	44	—	
	*Escherichia coli*	1,410	—	
	*Staphylococcus aureus*	44	—	

MIC, minimum inhibitory concentration; MBC, minimum bactericidal concentration, — = not determined, a = resistant bacteria strains, MDR, multi-drug resistant; CNS, central nervous system; CVC, central venous catheter; VRE, vancomycin-resistant *enterococcus*; ERY, erythromycin; TET, tetracycline; AMP, ampicillin; GEN, gentamicin; STR, streptomycin; TEIC, teicoplanin; VRSA, vancomycin-resistant *S. aureus*.

Auranofin is a trialkylphosphine gold complex approved for the treatment of RA (Harbut et al., 2015; Cassetta et al., 2014; Mingh, 2007) that has been in clinical use since 1985 (Shaw, 1999). Although the knowledge of the use of gold complexes in clinical settings is not new, the interest in them and their derivatives has risen in recent years due to their broad-spectrum antimicrobial activities and unique modes of action (Ratia et al., 2022). According to the literature data, auranofin is an anti-inflammatory agent most frequently studied for its antimicrobial activity, which produced the strongest *in vitro* growth inhibitory effect against *S. agalactiae, S. pneumoniae, S. aureus, S. epidermidis B. subtilis, C. difficile, E. faecalis, E. faecium,* and *M. tuberculosis* with MIC ranging from 0.0015 to 5 mg/L (Thangamani et al., 2016a; Thangamani et al., 2016b; Cassetta et al., 2014; Harbut et al., 2015; Abdelkhalek et al., 2019; Abdelkhalek et al., 2018; Abutaleb and Seleem, 2020a; Abutaleb and Seleem, 2020b). More importantly, the drug also showed potent activity against *S. aureus,* including drug-resistant clinical isolates, vancomycin-intermediate *S. aureus* (VISA), vancomycin-resistant *S. aureus* (VRSA) and methicillin-resistant *S. aureus* (MRSA), (MIC ranging from 0.0625–1.357 mg/L), the principal bacterial agent responsible for the most severe forms of MSKIs, such as osteomyelitis and septic arthritis (Cassetta et al., 2014; Harbut et al., 2015; Thangamani et al., 2016b; Ferretti et al., 2025; Quadros Barse et al., 2024; Chiaverini et al., 2022; Tong et al., 2015). Despite the broader range of MICs observed for *S. aureus*, the results adequately characterise its *in vitro* susceptibility to auranofin because, in seven independent studies, a total of 39 strains were tested, including clinical isolates and resistant strains. Auranofin also exhibited anti-biofilm properties as it reduced *S. aureus* biofilm mass by >60% at a concentration of 1 mg/L, which indicates its anti-biofilm activity (Thangamani et al., 2016b). Studies on the drug’s antimicrobial mechanism of action showed that it inhibits the bacterial thioredoxin reductase enzyme, which protects Gram-positive bacteria such as *S. aureus* against reactive oxidative species (Harbut et al., 2015; Liao et al., 2017).

The antimicrobial activity of aspirin was also investigated in a number of studies (Al-Bakri et al., 2009; Chan et al., 2017; Ozturk et al., 2021). Aspirin (also known as acetylsalicylic acid), a non-selective COX inhibitor used in managing acute MSKI-related pain, belongs to the salicylic acid derivatives (Ornelas et al., 2017; Kowalski and Stevenson, 2013; Davis et al., 2022). Research findings showed that this NSAID exhibited broad-spectrum antimicrobial activity against Gram-positive (*B. cereus*), Gram-negative (*E. aerogenes, K. pneumoniae, E*. *coli, P. aeruginosa, S. choleraesuis*) bacteria and yeast (*C. albicans*) with MIC of ≥1.2 mg/L (Al-Bakri et al., 2009; Chan et al., 2017). In addition, it exerted a moderate bactericidal effect against methicillin-susceptible *S. aureus* (MSSA) ATCC 25923 and MRSA ATCC 33591 at an MBC of 2.5 mg/L (Chan et al., 2017). At the same time, it showed weak MBC activity against MRSA clinical isolates, *B. cereus,* Gram-negative bacteria such as *S. choleraesuis, E. aerogenes, K. pneumoniae, P. aeruginosa, E. coli* and yeast (*C. albicans*) at MBC ≥4.8 mg/L (Chan et al., 2017; Al-Bakri et al., 2009). Interestingly, aspirin exerted a similar antimicrobial effect against MSSA (ATCC25923) and MRSA (ATCC 33591) strains, including resistant clinical isolates with a MIC of 2.5 mg/L (Chan et al., 2017). However, there were some differences in the effect of aspirin against *E. coli* strains. For example, the MIC obtained when aspirin was tested against *E. coli* ATCC 8739 and *E. coli* ATCC 25922 was 1.2 mg/L and 5 mg/L. Similarly, the susceptibility of *P. aeruginosa* ATCC 9027 and *P. aeruginosa* ATCC 10145 to aspirin were with MIC of 2.03 mg/L and 5 mg/L, respectively (Chan et al., 2017; Al-Bakri et al., 2009). Aspirin also exhibited an antibiofilm activity against *E. coli, P. aeruginosa,* and *C. albicans*, causing a concentration-dependent reduction of the viable bacteria count. The kinetics of antibiofilm effect results showed that an exposure time of 4 h caused a percentage reduction of 98.23, 94.25 and 93.61 in the viable counts of *P. aeruginosa*, *E. coli* and *C. albicans* biofilms, respectively. Aspirin’s minimal biofilm eradication concentration values against the established biofilms ranged between 1.35 and 3.83 mg/L (Al-Bakri et al., 2009). The antibiofilm activity of aspirin against certain bacteria (e.g. *P. aeruginosa*) is believed to stem from its ability to inhibit quorum sensing by downregulating key quorum-sensing genes (*lasI, lasR, rhlR, pqsA, pqsR*). This disruption leads to reduced production of biofilm, adhesins, and toxins (El-Mowafy et al., 2014). Moreover, another study found that aspirin downregulates *alg*D expression, increasing bacterial susceptibility to antibiotics (Tabatabaeifar et al., 2022). The *alg*D operon in *P. aeruginosa* regulates alginate synthesis, thereby promoting biofilm formation and bacteria resistance to phagocytosis and antibiotics (Blanco-Cabra et al., 2020; Powell et al., 2018). Aspirin also exhibits antibiofilm activity against *S. aureus* by the inhibition of *agrA*-regulated virulence genes and downregulating biofilm-associated genes such as *icaA* and *fnbA* (Tabatabaeifar et al., 2022). Notably, aspirin suppresses *icaA* expression, which is crucial for producing polysaccharide intercellular adhesin, a key structural component of *S. aureus* biofilms (Cramton et al., 1999). Moreover, clinical evidence suggests that aspirin therapy reduces the risk of *S. aureus*-induced bacteremia; therefore, it is recommended for postoperative treatment to decrease graft-related infections emanating from coagulase-negative staphylococci such as *S. epidermidis,* which is implicated in osteomyelitis and septic arthritis (Sedlacek et al., 2007; Demirag et al., 2007). However, studies have shown that aspirin slightly increases fluoroquinolone resistance in ciprofloxacin-susceptible and resistant *S. aureus* strains (Gustafson et al., 1999). It enhances fluoroquinolone resistance by inducing the *S. aureus* multiple antibiotic resistance operon, which increases the production of the *S. aureus* NorA efflux pump, consequently reducing fluoroquinolone accumulation (Ohshita et al., 1990). Additionally, Verma et al. (2018) demonstrated that exposure of planktonic *E. coli* to aspirin could increase resistance to ciprofloxacin and tetracycline.

Ibuprofen, another NSAID possessing antimicrobial activity, is an essential drug developed in the 1960s for treating RA. It is currently one of the most used non-prescription drugs globally (Davies, 1998; Oliveira et al., 2019), which belongs to the aryl propionic acid class of compounds (Kowalski and Stevenson, 2013). Chan et al. (2017) evaluated the antibacterial activity of ibuprofen against several bacteria. Findings showed that the drug mostly inhibited the growth of *B. cereus*, followed by MRSA and MSSA, with MIC ranging from 0.625 to 2.5 mg/L. Further bactericidal studies confirmed the potency of this compound against *B. cereus* and *S*. *aureus* with an MBC range of 2.5–5.0 mg/L (Chan et al., 2017). The drug also showed bactericidal activity against the MRSA clinical isolates (MBC ≥5 mg/L), thus exhibiting both bacteriostatic and bactericidal effects against *B. cereus* and *S. aureus* (Chan et al., 2017). In another study, Al-Janabi. (2010) demonstrated that *P*. *yeei* and *S. aureus* were the most susceptible pathogens to ibuprofen and obtained a lower MIC of 1.25 mg/L against *S. aureus.* The investigation of ibuprofen’s antimicrobial mechanism of action on *S. aureus* using potassium iodide uptake and intracellular K^+^ release tests showed evidence of cytoplasmic membrane destabilisation and disruption (Oliveira et al., 2019). By monitoring the number of colony-forming units and growth kinetics, Shah et al. (2018) demonstrated that ibuprofen reduced the growth rate of *P. aeruginosa.* This pathogen causes life-threatening MSKIs such as osteomyelitis. Similarly, Dai et al. (2019) proved that ibuprofen inhibited *P. aeruginosa* biofilm formation. Ibuprofen demonstrated antibiofilm effects by interfering with quorum-sensing signalling molecules (*lasI, lasR, rhll, rhlR, pqsA*, and *pqsR*) in *P. aeruginosa*, thereby reducing the expression of biofilm-associated genes. Additionally, ibuprofen reduced the release of pyocyanin, rhamnolipid and protease—key virulence factors regulated in *P. aeruginosa* by quorum-sensing (Dai et al., 2019). Also, a recent study demonstrated that ibuprofen significantly decreased the transcription level of *algD* in *P. aeruginosa* and *icaA* in *S. aureus* (Tabatabaeifar et al., 2022). Since quorum sensing inhibitors act to directly prevent biofilm formation and limit the production of virulence factors, they are being considered as a novel strategy for treating *P. aeruginosa* infections (Le Berre et al., 2006). Thus, the antibiofilm and anti-quorum sensing activity of ibuprofen indicates that it can be a candidate drug for the treatment of clinical infections caused by *P. aeruginosa* (Dai et al., 2019). Besides its antibacterial action, ibuprofen also inhibited the growth of several Candida strains with a MIC ranging from 1 to 3 mg/L (Pina-Vaz et al., 2000).

Diclofenac is among the most used NSAIDs for reducing fever, pain, and inflammation, especially in patients with arthritis (Zhang et al., 2021; Hamed et al., 2021). The drug, which belongs to the heteroaryl acetic acid class of NSAIDs (Kowalski and Stevenson, 2013), produced antibacterial properties against several bacteria (Chan et al., 2017). Among them, MRSA and *B. cereus* were the most susceptible pathogens, with MIC ranging from 0.3125 to 2.5 mg/L. However, Dastidar et al. (2000) demonstrated that diclofenac inhibited the growth of *S. aureus* at a much higher MIC of 50 mg/L. Variations in the MIC values observed may be attributed to the difference in the susceptibility of standard strains and clinical isolates tested. Diclofenac was also bactericidal against *B. cereus* and MRSA ATCC 33591 with an MBC of 2.5 mg/L (Chan et al., 2017). Notably, this agent displayed a low resistance rate compared to antibiotics, such as daptomycin and vancomycin (Humphries et al., 2013; Zhang et al., 2021). A study that investigated the anti-virulence effect of diclofenac against multi-drug resistance MRSA clinical isolates demonstrated a significant reduction in biofilm formation. Also, remarkable inhibition of hemolysin activity was observed. In addition, diclofenac has inhibitory activity against staphyloxanthin production and downregulated MRSA virulence genes, including *SarA, Hla, FnbA, IcaA, SigB, CrtM* and *AgrA,* which is considered a quorum sensing regulatory gene of *S. aureus* and plays a role in the upregulation of superantigens, cytotoxins, and secreted enzymes (Abbas et al., 2020). Similarly, Elmesseri et al.(2023) showed that diclofenac potently inhibited the synthesis of staphyloxanthin, a key virulence factor for the survival of MRSA against host innate immunity. Additionally, treated cells revealed a significant downregulation of virulence genes responsible for staphyloxanthin synthesis, such as *crtM, crtN* and global transcriptional regulator *sigB*, along with the *Hla* gene (Elmesseri et al., 2023). Investigation of proteomic alterations in MRSA showed that diclofenac alters the pathways associated with β-lactams resistance, energy metabolism, and peptidoglycan biosynthesis (Zhang et al., 2021).

Celecoxib is a selective COX-2 inhibitor approved for treating RA (Silverstein et al., 2000; Krasselt et al., 2021; Dinescu et al., 2021). It belongs to the pyrazole class of drugs and has several clinical applications due to its antimicrobial, anti-inflammatory, and analgesic properties (Kucukguzel and Senkardes, 2015). Thangamani et al. (2015) investigated celecoxib’s antibacterial effect against several multidrug-resistant and Gram-negative bacteria strains. The drug showed a moderate antibacterial effect against all the Gram-positive bacteria tested, including *B. anthracis*, *B. subtilis*, MRSA, VRSA, *L*. *monocytogenes,* MRSA, and VISA clinical isolates (MIC = 16–32 mg/L). In contrast, the drug was inactive against all the Gram-negative bacteria. Chiu et al. (2012) and Gajdacs and Spengler (2019) demonstrated the anti-staphylococcal effect of this drug against *S. aureus* and *S. epidermidis* with MIC ranging from 15 to 32 mg/L*.* In addition, Okpala et al. (2024) recently showed that celecoxib inhibited the growth of *S. aureus*, including the clinical isolates at MIC ranging from 32 to 64 mg/L. Regarding the drug’s antimicrobial mechanisms of action, Thangamani et al. (2015) demonstrated that celecoxib inhibits RNA, DNA, and protein synthesis in *S. aureus*.

Diacerein, a semisynthetic anthraquinone derivative that inhibits interleukin-1β is commonly used as a slow-acting drug to treat joint diseases such as osteoarthritis (Bruneton, 1999; Fidelix et al., 2014; Pavelka et al., 2016). Zhang et al. (2019) examined the antibacterial activity of diacerein against several Gram-positive cocci isolated from bacterial keratitis patients. Their results showed that the most susceptible bacteria to this agent were *S. epidermidis*, *S. xylosus*, *S. intermedius,* and *S. haemolyticus,* with MIC ranging from 1 to 16 mg/L. In a separate study, Nguon et al. (2013) also demonstrated the anti-staphylococcal effect of diacerein against several *S. aureus* strains, including MSSA and MRSA, with MIC values ranging from 16 to 43 mg/L.

Based on the literature data, the NSAIDs produced a stronger effect against Gram-positive (MICs = 0.0015–2000 mg/L, MBCs = 2–40 mg/L) than towards Gram-negative (MICs = 1.2–4,000 mg/L, MBCs = 4.8–500 mg/L) bacteria, which suggests a higher susceptibility of Gram-positive strains to these agents. The higher resistance of Gram-negative bacteria to anti-inflammatory drugs may be due to the permeability barrier conferred by their outer membrane (Thangamani et al., 2015) and the presence of more effective multi-drug resistance efflux pump systems (Laudy, 2018; Quadros Barse et al., 2024). For example, the antimicrobial activity of celecoxib was restored when the outer membrane barrier was compromised with the antibiotic colistin (Thangamani et al., 2015). In another study, the deficiency of TolC proteins in the outer membrane of *E. coli* increased its susceptibility to auranofin (Quadros Barse et al., 2024). Additionally, deleting the AcrAB efflux pump in *E. coli* restored their susceptibility to celecoxib treatment (Thangamani et al., 2015).

### 2.2 *In vivo* experiments

Preclinical research with animal models has been the gold standard for decades, and it is an important criterion for determining the safety and efficacy of drugs before introducing them to the market (Mahalmani et al., 2023). However, it has also been observed that effects found in animal models cannot always be translated to the clinic (Martic-Kehl et al., 2012), thereby questioning the relevance of preclinical studies. For example, Langley (2009) stated that less than 50% of animal studies predicted human outcomes sufficiently. Additionally, animal models are poor predictors of drug safety in humans. Consequently, humans have been exposed to toxicity in the clinical testing of drugs that were believed to be safe in animal studies (Van Norman, 2019). For example, an analysis of 2, 366 drugs showed that results obtained from animal testing (e.g. rat, mouse and rabbit models) were inconsistent predictors of toxic responses in humans (Bailey et al., 2014). Since the drugs analysed in this review are currently in use in clinical settings, having undergone clinical trials and passing extensive toxicity and safety evaluations, they carry lower risks of adverse reactions (March-Vila et al., 2017). The *in vivo* studies were conducted to evaluate the antimicrobial effects of five anti-inflammatory drugs, namely auranofin, aspirin, ibuprofen, celecoxib, and diclofenac against seven pathogenic bacteria (*C. difficile*, *E. faecium*, *E. faecalis*, *M. tuberculosis*, *P. aeruginosa*, *S. aureus*, and *S. enterica* serotype Typhimurium) as well as the fungus *Histoplasma capsulatum*. These studies were performed in various infected mouse strains (BALB/c, C3HeB/FeJ, C57BL/6, CD1, and Swiss albino mice). Among the tested drugs, auranofin and ibuprofen were the most frequently assessed for their antimicrobial activity, while *M. tuberculosis* was the most commonly studied bacterial pathogen. Detailed data on the *in vivo* antimicrobial activity of these anti-inflammatory drugs are presented in Table 2.

**TABLE 2:** *In vivo* antimicrobial activity of anti-inflammatory drugs.

Compound	Microorganism	Animal model	Agent dosage	Result	References
Aspirin	*Mycobacterium tuberculosis*	BALB/c mice	20 mg/kg/day of aspirin, +150 mg/kg/day of pyrazinamide, for 4 weeks (orally), ten animals (5 per treatment and control group)	drug combination completely eradicated infection from the spleen of 40% of the treated animals when compared with the control group	Byrne et al. (2007)
		C3HeB/FeJ mice	3 mg/kg/day of aspirin was administered for 2 weeks in 24 animals (12 per therapeutic and control groups)	the drug showed >90% survival rate in the treated groups compared with 70% in the control groups	Kroesen et al. (2018)
Auranofin	*Clostridioides difficile*	C57BL/6 mice	0.125, 0.25, and 0.5 mg/kg/day (groups I, II, III, respectively) of auranofin, 10 mg/kg/day (group IV) of vancomycin (positive control), the negative control group (vehicle = 10% DMSO in PBS, group V) for 5 days (orally), 25 animals (5 per treatment and control groups)	groups I and II of drug-treated animals showed 100% and 80% survival rates; group III only showed 40% survival rate. The untreated group (vehicle) showed only about 20% survival rate	Abutaleb and Seleem (2020b)
	*Enterococcus faecium* ^a^ (VRE)	C57BL/6 mice	0.5 mg/kg/day of auranofin (group I), 10 mg/kg/day each of linezolid and ramoplanin (groups II and III, respectively), or phosphate-buffered saline control (group IV) for 8 days (orally), 20 animals (5 per treatment and control groups)	the bacterial burdens in the drug-treated animals were reduced by 98% and 99% after 3 and 5 days, respectively. No reduction was observed in the untreated control	Abdelkhalek et al. (2018)
		BALB/c mice	0.125,0.25 and 0.5 mg/kg/day (groups I, II, and III, respectively) of auranofin, 20 mg/kg/day (group IV) of linezolid (orally), 0.0625, 0.125, and 0.25 mg/kg/day (groups V, VI and VII, respectively) of auranofin (subcutaneously), two control groups (orally and subcutaneously), for 4 days, 45 animals (5 per treatment and control groups)	the drug-treated groups I, V, and VI showed 100% survival from bacterial infection. About 30% of the animals in the control groups survived	Abutaleb and Seleem (2020a)
	*Staphylococcus aureus* ^a^ (MRSA)	CD1 mice	0.012 and 0.12 mg/kg/day of auranofin (groups I and II, respectively) for 7 days intraperitoneally), 24 animals (8 per treatment and control group)	37% and 50% of the drug-treated animals in groups I and II survived. All the animals in the control group died by day 4	Harbut et al. (2015)
		BALB/c mice	0.125 and 0.25 mg/kg/day (groups I and II, respectively) of auranofin, 25 mg/kg/day (group III) of linezolid for 3 days (orally), 40 animals (10 per treatment and control group)	the survival rates of the animals in the drug-treated groups I and II were 40% and 80%, respectively. <40% of animals in the control group survived	Thangamani et al. (2016a)
Celecoxib	*Histoplasma capsulatum*	C57BL/6 mice	1 mg/kg/0.5 mL/day of celecoxib (group I), water-treated group (group II), non-infected control group (group III), for 30 days (orally), 19 animals (7 per treatment and water-treated control, 5 for non-infected control, respectively)	the drug caused a 70% survival rate in the treated animals when compared with the control groups	Pereira et al. (2013)
	*Staphylococcus aureus* ^a^ (MRSA)	BALB/c mice	20 mg/kg 2x/day of 1% and 2% celecoxib cream (topically, groups I and II), 20 mg/kg 2x/day of 2% fusidic acid (topically, group III), 25 mg/kg 2x/day of clindamycin (orally, group IV) and control group (20 mg petroleum jelly) for 5 days, twenty-five animals (5 per treatment and control groups)	1% and 2% of the drug significantly lowered the bacterial burden in the drug-treated animals by 72% and 87% compared with the control group	Thangamani et al. (2015)
Diclofenac/Diclofenac sodium	*Mycobacterium tuberculosis*	Swiss albino mice	10 mg/g/day of diclofenac (orally),150 mg/g/day of streptomycin (subcutaneously), 10 mg/g/day of diclofenac (orally) + 150 mg/g/day of streptomycin (subcutaneously) for 4 weeks, 50 animals (10 per treatments, day one control for baseline values and untreated control groups)	animals treated with diclofenac or streptomycin and combinations showed a 60, 70% and 100% survival rate. All the animals in the control groups died	Dutta et al. (2007a)
	*Salmonella enterica* serotype Typhimurium	Swiss albino mice	15,30 and 60 mg/g (groups I, II and III, respectively) of diclofenac for 100 h (intraperitoneally), 120 animals (20 per treatment group and 60 for the control group)	the drug-treated groups I, II and III showed a 65%, 70% and 75% survival rate. All the animals in the control group died	Dutta et al. (2007b)
Ibuprofen	*Mycobacterium tuberculosis*	BALB/c mice	20 mg/kg/day of ibuprofen, +150 mg/kg/day of pyrazinamide (5 days per week) for 4 weeks (orally), ten animals (5 per treatment and control groups)	the drug combination completely eradicated infections from the spleen of 60% of the animals compared to the control group	Byrne et al. (2007)
		C3HeB/FeJ mice	80 mg/kg/day of ibuprofen for 1 week (orally), 36 animals (18 per treatment and control groups)	the drug-treated animals showed an 80% cure of infected lung areas compared to 21% in the control group	Vilaplana et al. (2013)
	*Pseudomonas aeruginosa*	C57BL/6 mice	0.75 mg/kg of ibuprofen at 8 hrs. Intervals until 64th hrs. (orally), 27 animals (13 and 14 for the treatment and control groups, respectively)	the drug-treated group showed a 92% survival rate compared to 57% in the control group	Shah et al. (2018)

a = resistant bacteria strains, VRE, vancomycin resistant *Enterococcus faecium*.

#### 2.2.1 Auranofin

Among all the anti-inflammatory drugs tested for *in vivo* antimicrobial effects, auranofin produced the effect at the lowest doses administered to the experimental animals. In the experiment with mice infected with vancomycin-resistant *E*. *faecium* (VRE), the animals showed a significantly higher survival rate (100%) compared to the control group (30%) when administered subcutaneously with 0.0625 mg/kg/day and orally with 0.125 mg/kg/day of auranofin (Abutaleb and Seleem, 2020a). In comparison, the subcutaneous administration of glycopeptide antibiotic teicoplanin at 40 mg/kg/day increased the survival rate only in two of five mice strains infected with VRE (Song et al., 2008). In another study performed with mice challenged with *E. faecium,* the animals experienced 98% and 99% reduction in bacterial burdens after oral treatment with 0.5 mg/kg/day of auranofin for 3 and 5 days, respectively. On the other hand, no reduction in infection was observed in the untreated control group. Interestingly, auranofin outperformed linezolid, a synthetic oxazolidinone antimicrobial drug approved for treating VRE infections, which failed to reduce the VRE burden in the mice within 3 days period and was only able to reduce bacterial burden by 52% after 5 days of treatment (Abdelkhalek et al., 2018). In another study, the antimicrobial action of auranofin was investigated against mice infected with *S. aureus* (Thangamani et al., 2016a). After administering 0.25 mg/kg/day of the drug orally to the mice, the animals recorded an 80% survival rate compared to <40% in the control group. Similarly, oral treatment with antibiotic linezolid achieved a high survival rate in mice. However, this was at a much higher dose of 25 mg/kg/day (Thangamani et al., 2016a). The intraperitoneal administration of 0.12 mg/kg/day and 0.012 mg/kg/day of auranofin to mice infected with *S. aureus* for 7 days only caused moderate to weak effects, with the animals showing 50% and 37% survival rates, respectively. All the animals in the control group died by day 4 (Harbut et al., 2015). Auranofin at low doses (0.125 mg/kg and 0.25 mg/kg) significantly protected mice against *C. difficile* infection (CDI) with a 100% and 80% survival rate. At these doses, the agent prevented CDI recurrence in the animals compared to vancomycin, which had a similar effect but at a higher dose of 10 mg/kg (Abutaleb and Seleem, 2020b). These results demonstrated auranofin’s strong *in vivo* antimicrobial effect against pathogenic bacteria such as *S. aureus*, *E. faecium,* and *C. difficile*. Moreover, evidence from toxicology studies shows that auranofin is safe. For example, no significant histopathologic lesions were observed on porcine skin after exposure to 1%, 2%, and 3% auranofin (topically) for 4–14 days. In addition, no systemic toxicity was observed in these pigs after exposure to this agent (Mohammad et al., 2021). Therefore, auranofin, an FDA-approved drug with a long history of clinical use, holds promise as a dual-action therapy with fewer side effects (Ito et al., 2022).

#### 2.2.2 Ibuprofen

Shah et al. (2018) investigated the *in vivo* antimicrobial action of ibuprofen against mice infected with *P. aeruginosa*. After the oral administration of 0.75 mg/kg of ibuprofen, the animals showed a 92% survival rate compared to the control group (57%). In comparison, the intraperitoneal administration of either ceftazidime (1,000 mg/kg) or ciprofloxacin (100 mg/kg) every 8 h for 7 days to mice with *P. aeruginosa* infection resulted in 40%–60% animal survival (Song et al., 2012). When 80 mg/kg/day of the drug was orally given to the mice infected with *M. tuberculosis*, the animals experienced an 80% cure rate in their infected lungs. At the same time, the control group showed a 21% survival rate (Vilaplana et al., 2013). In addition, the oral administration of the combination of 20 mg/kg/day of ibuprofen with 150 mg/kg/day of pyrazinamide resulted in the eradication of *M. tuberculosis* infection from the spleen of 60% of the treated mice (Byrne et al., 2007).

#### 2.2.3 Aspirin

The antimicrobial activity of aspirin alone or in combination with standard anti-TB drugs was investigated in an experimental murine model of acute TB. The treatment of TB-infected mice with 3 mg/kg/day of aspirin resulted in >90% of the animals surviving compared to about 70% in the control group within the same period (Kroesen et al., 2018). Similarly, mice treated with the standard antibiotic combination known as RIMSTAR also achieved a high survival rate in mice. Nevertheless, this was attained at high RIMSTAR doses comprising rifampicin 150 mg, isoniazid 75 mg, pyrazinamide 400 mg, and ethambutol 275 mg (Kroesen et al., 2018). In another study, mice infected with TB were investigated to determine the combined antimicrobial effect of anti-tuberculosis agent pyrazinamide and aspirin. This combination, which involved 20 mg/kg/day of aspirin and 150 mg/kg/day of pyrazinamide, completely eradicated TB infection from the spleen of 40% of the treated mice after 1 month of oral administration. When administered alone orally to mice infected with *M. tuberculosis*, pyrazinamide caused a reduction of infection in the lung and spleen of the mice at a dose of 150 mg/kg per day after 1 month of treatment (Byrne et al., 2007). Toxicity evaluations of aspirin revealed varying safety profiles across different animal species. For example, rats and mice exhibited different responses when fed diets containing aspirin at concentrations of 0, 0.3, 0.6, and 1.2% for one and 4 weeks. The rats group exhibited dose-dependent haemorrhagic anaemia and death, while no apparent signs of haemorrhage were observed in mice (Takahashi and Hiraga, 1985). The toxicity of aspirin to cats is due to the poor metabolism of the drug in these animals compared to humans (Bell, 2019). Unlike humans, cats lack the key enzymes responsible for aspirin clearance (Court, 2013). Despite this, aspirin remains widely used and, when taken correctly, offers significant benefits such as reducing the risk of heart attacks, stroke and blood flow disorders in individuals with cardiovascular disease (FDA, 2019).

#### 2.2.4 Diclofenac

When *M. tuberculosis-*infected mice were treated with 10 mg/g of diclofenac (orally), they showed a 60% cure rate. A combined co-administration of diclofenac at 10 mg/g/day (orally) and streptomycin at 150 mg/g/day (subcutaneously) resulted in a 100% survival rate in these animals. In addition, this combination treatment regimen statistically caused significantly fewer bacteria in the lungs and spleen of these mice compared with those receiving streptomycin alone (Dutta et al., 2007a). This suggests that diclofenac synergistically enhances the efficacy of streptomycin. Also, a survival rate of 65% was attained when 15 mg/g/day of diclofenac was administered intraperitoneally to mice suffering from salmonella infection. Subsequent administration of this drug at higher doses of 30 and 60 mg/g/day increased the animal’s survival rate to 70% and 75%, respectively. In contrast, all the animals in the control group died (Dutta et al., 2007b).

#### 2.2.5 Celecoxib

Celecoxib demonstrated *in vivo* antibacterial activity in a mouse model of MRSA skin infection. The topical application of 1 and 2% celecoxib cream (20 mg 2x/day) significantly reduced the bacterial burden in infected mice by 72% and 87%, respectively (Thangamani et al., 2015). Besides their antibacterial activity, celecoxib also demonstrated anti-fungal action by causing a 70% survival rate in mice with lethal *H. capsulatum* infection after oral treatment with 1 mg/kg/0.5/day of celecoxib (Pereira et al., 2013). Moreover, preclinical animal studies suggest that celecoxib has a favourable safety profile in rats, as it did not cause gastrointestinal mucosal damage (Altinkaynak et al., 2003; Li et al., 2003). Also, it is well tolerated in humans for long-term oral use with fewer gastrointestinal side effects compared to traditional NSAIDs (Kishore et al., 2016; Sozer et al., 2011). Nevertheless, there has been reported exacerbation of inflammation-associated colonic injury in rats treated with celecoxib (Zhang et al., 2004). Additionally, the agent has been linked with an elevated risk of heart-related conditions in humans, especially when taken at substantially high doses beyond those recommended for arthritis treatment (FitzGerald, 2003; Howes, 2007).

### 2.3 Clinical trials

In general, the number of clinical trials on the antimicrobial effects of anti-inflammatory drugs is very low and available studies are focused mainly on uncomplicated UTIs. NSAIDs, namely aceclofenac, diclofenac, and ibuprofen, were tested in clinical trials with patients diagnosed with uncomplicated UTIs and cellulitis (Gagyor et al., 2015; Bleidorn et al., 2010; Vik et al., 2018; Dall et al., 2005; Davis et al., 2017; Kronenberg et al., 2017; Ko et al., 2018). Among these drugs, ibuprofen was the most frequently tested in double-blind, randomised, controlled trials with UTI patients. Detailed data on the efficacy of anti-inflammatory drugs against UTIs and cellulitis are summarised in Table 3.

**TABLE 3:** Clinical trials demonstrating the effectiveness of anti-inflammatory drugs in symptom resolution among UTI and cellulitis patients.

Compound	Study design	Agent dose	NI	ICS	Age	Results	References
Aceclofenac	prospective open-labeled and randomised controlled pilot study	100 mg of cefpodoxime 2x/day (group I), 100 mg of cefpodoxime +100 mg of aceclofenac 2x/day (group II), for 3 days (orally)	55 (28 and 27 for groups I and II, respectively)	uncomplicated cystitis	≥18	76% of patients in both groups experienced symptom resolution, but patients in group II experienced faster resolution than group I patients	Ko et al. (2018)
Diclofenac	a double-blind, randomised, controlled trial	75 mg of diclofenac 2x/day (group I), 400 mg of norfloxacin 2x/day (group II) for 3 days (orally)	253 (133 and 120 for groups I and II, respectively)	uncomplicated UTI	18–70	54% and 80% of the patients had their symptoms resolved by day 3 in groups I and II, respectively. 62% and 98% of patients in groups I and II used antibiotics for 30 days post-treatment	Kronenberg et al. (2017)
Ibuprofen	a double-blind, randomised, controlled pilot trial	400 mg of ibuprofen 3x/day (group I), 250 mg of ciprofloxacin 2x/day (+placebo) (group II), for 3 days (orally)	79 (40 and 39 for groups I and II, respectively)	uncomplicated UTI	18–85	58% of patients in group I were symptoms-free compared to 51% in group II	Bleidorn et al. (2010)
	a double-blind, randomised, controlled trial	400 mg of ibuprofen 3×/day (group I) for 3 days, a single dose of 3 g of fosfomycin (group II), with their respective placebos (orally)	484 (241 and 243 for groups I and II, respectively)	uncomplicated UTI	18–65	46% of the patients in group II were symptomatic for more than 2 days compared to 36% in group I	Gagyor et al. (2015)
	a double-blind, randomised, controlled trial	600 mg of ibuprofen 3x/day (group I), 200 mg of pivmecillinam 3x/day (group II), for 3 days (orally)	383 (194 and 189 for groups I and II, respectively)	uncomplicated UTI	18–60	39% of patients in group I recovered from UTI compared to 73% in group II 53% of patients in group I recovered without antibiotics treatment	Vik et al. (2018)
	a double-blind, randomised, controlled trial	200 mg of ibuprofen 3x/day (group I), placebo 2 × 3/day (group II) for 5 days (orally)	51 (25 and 26 for groups I and II, respectively)	uncomplicated cellulitis	18–80	80% of group I patients experienced a decline in inflammation compared to 65% in the placebo at 48 h	Davis et al. (2017)
	prospective pilot study	500 mg of cephalexin 4x/day (group I) for 10 days, 500 mg of cephalexin 4x/day for 10 days +400 mg of ibuprofen every 6 hrs. (group II) for 5 days (orally)	64 (33 and 31 for groups I and II, respectively)	cellulitis	—	100% of patients in group II recovered from cellulitis in ≤5 days, while 24% of patients in group I needed 6–7 days to recover	Dall et al. (2005)

Age = in years, ICS, inclusion criteria symptoms; UTI, urinary tract infections; NI, number of individuals, — = not indicated.

Primary care physicians usually prescribe antibiotics for treating uncomplicated UTIs (Butler et al., 2017; Fahey et al., 2003), but NSAIDs have also been recommended as a first-line treatment option in women aged <65 years with suspected uncomplicated lower UTIs who experienced mild symptoms and as an alternative to antibiotics when the symptoms are moderate to severe (Scottish Intercollegiate Guidelines Network, 2020). Consequently, ibuprofen has been recommended for pain management and symptom relief in patients with lower UTI, such as cystitis (National Institute for Health and Care Excellence, 2018; Bettcher et al., 2021). Correspondingly, ibuprofen is also the most studied NSAID in clinical trials, resulting in positive outcomes for UTI patients. For example, the administration of 400 mg 3x/day of this agent to UTI patients for 3 days resulted in 58% and 75% rates in symptom resolution by days 4 and 7, respectively, compared to 51% and 60% in those treated with 250 mg of ciprofloxacin 2x/day (Bleidorn et al., 2010). In another study, only a lesser percentage (36%) of patients administered 400 mg of ibuprofen 3x/day for 3 days experienced UTI symptoms over 2 days compared with 46% of those treated with a single dose of 3 g fosfomycin. Also, cases of recurrent UTI were reported more (23%) by women assigned to the fosfomycin group compared to the ibuprofen group with 17%. Additionally, the initial treatment of patients with ibuprofen caused a 67% reduction in antibiotic use. However, women using ibuprofen were more likely to report a higher burden of symptoms over the first 7 days after the start of their treatment compared with those using only fosfomycin (Gagyor et al., 2015). Although ibuprofen at 600 mg 3x/day was inferior to pivmecillinam at 200 mg 3x/day in treating uncomplicated UTIs, 53% of the patients treated with ibuprofen recovered without antibiotic use after 4 weeks of follow-up (Vik et al., 2018). Also, ibuprofen seems relatedly safe in animal models. For example, a study on the gastrointestinal and renal safety of piglets administered with 5 mg/kg of ibuprofen 3x/day for 5 days demonstrated that this agent was well-tolerated because no severe lesions or significant histological changes were detected in their stomach or kidneys (Millecam et al., 2019). However, caution is advised when using ibuprofen, as its administration may lead to a dose-dependent increase in gastrointestinal permeability (Khazaeinia and Jamali, 2000). Thus, as a non-COX inhibitor, it can compromise gastric mucosal integrity, potentially leading to intramucosal haemorrhages, though this effect is typically observed with chronic use (Ershad et al., 2024).

A separate study investigating the superiority of 75 mg 2x/day diclofenac over 400 mg 2x/day norfloxacin in UTI patients after 3 days showed that diclofenac was less effective than norfloxacin; however, those patients on diclofenac were 37% less likely to receive antibiotic treatment until day 30 post-randomisation (Kronenberg et al., 2017). Antibiotics used by UTI patients account for 10%–20% of all the antibiotics prescribed in ambulatory care (Melnyk et al., 2024). Furthermore, many studies have shown that there is a clear Incorrelation between antibiotic consumption and increased drug resistance in uropathogens such as *E.coli* Therefore, reducing the prescription and use of antibiotics for UTI treatment could help reduce the risk of antibiotic resistance in the population (Kronenberg et al., 2017). When considering side effects, diclofenac is generally safe, with its benefits outweighing potential risks. For example, it is well-tolerated for topical treatment of musculoskeletal conditions (European Medicine Agency, 2013; Taylor et al., 2011). Nevertheless, animal studies show evidence of toxicity, including gastric damage in rats at high doses (Aycan et al., 2018). Also, in humans, there is a small risk of heart attack or stroke with prolonged systemic exposure to high doses (150 mg daily) of this agent (European Medicine Agency, 2013). Another study evaluated the efficacy of combining 100 mg of aceclofenac (an analogue of diclofenac) 2x/day with 100 mg of cefpodoxime for the treatment of uncomplicated cystitis (a form of UTI) compared to 100 mg of cefpodoxime 2x/day for 3 days. Results showed that 76% of the patients in both groups experienced symptom resolution. However, patients in the combination group had a faster symptom resolution than those administered with cefpodoxime alone (Ko et al., 2018).

Cellulitis is a diffuse spreading infection with inflammation of the deeper dermis and subcutaneous fat. It is caused mainly by *S. aureus* (Horseman and Bowman, 2013). A study that compared the efficacy of 200 mg 3x/day of ibuprofen to a placebo 2 × 3/day for 5 days in treating uncomplicated cellulitis showed 80% inflammation regression in the ibuprofen-treated group compared to 65% in the placebo. (Davis et al., 2017). Additionally, administering a combination of 400 mg of ibuprofen every 6 hours for 5 days and 500 mg of cephalexin 4x/day for 10 days led to a total recovery of all the patients from cellulitis infection in 4–5 days. In contrast, only about 24% of patients treated with 500 mg of cephalexin 4x/day for 10 days recovered, which took 6–7 days, while 6% required 7 days or more to recover (Dall et al., 2005). Overall, these agents appear effective in alleviating symptoms in UTI patients.

## 3 Conclusion

In conclusion, the literature analysis of *in vitro*, *in vivo* and clinical data identified 18 anti-inflammatory drugs with antibacterial activities. Among three drugs (aceclofenac, diclofenac and ibuprofen) studied in clinical trials, ibuprofen was the most frequently tested agent, and its administration resulted in a higher rate of symptom resolution and reduced antibiotic use in UTI patients. Although there is no clear evidence about its antimicrobial effect from clinical trials, *in vivo* results showed that ibuprofen at a low dose significantly increased the survival rate in mice infected with *P. aeruginosa*. It also demonstrated potent *in vitro* growth and inhibitory effects against *B. cereus*, *E. coli*, MRSA and MSSA. For the remaining 15 drugs (auranofin, aspirin, bromfenac, carprofen, celecoxib, diacerein, flufenamic acid, flurbiprofen, indomethacin, meclofenamic acid, naproxen, nimesulide, sodium salicylate, tolfenamic acid and vedaprofen), only data from *in vitro* and *in vivo* experiments are available. Among these, auranofin caused a significantly higher survival rate in mice infected with vancomycin-resistant *E. faecium*, *S. aureus* and *C. difficile.* It also produced a strong *in vitro* growth-inhibitory effect against *S. agalactiae*, *S. pneumoniae*, *S. aureus*, *S. epidermidis*, *B. subtilis, C. difficile, E. faecalis, E. faecium,* and *M. tuberculosis.* Among other antimicrobially active anti-inflammatory drugs, aspirin showed a significant effect *in vivo* against *M. tuberculosis* and strong to moderate *in vitro* activity against *E. coli*, *B. cereus*, *P*. *aeruginosa*, *E. aerogenes*, *K. pneumoniae* and *S*. *choleraesuis*. Similarly, celecoxib produced significant *in vivo* effects when applied topically against MRSA but only moderate *in vitro* effects against *S. epidermidis*, *S. aureus* and *B. subitilis.* These data suggest that certain NSAIDs are promising drug candidates for the development of dual-action drugs for the potential treatment of TB, MSKIs, and UTIs. However, future clinical trials are required to evaluate the antibacterial efficacy of anti-inflammatory drugs in treating TB, MSKIs, and UTIs before they can be integrated into clinical practice. Additionally, future research is needed to investigate their dual mechanism of action—combining anti-inflammatory and antimicrobial effects. Moreover, exploring targeted drug delivery systems for these agents could further enhance their therapeutic potential by minimising systemic side effects or off-target exposure, especially in the treatment of complex infectious and inflammatory diseases such as osteomyelitis.
